# Ezetimibe: An Unusual Suspect in Angioedema

**DOI:** 10.1155/2020/9309382

**Published:** 2020-02-27

**Authors:** Tiffany Lu, Tarundeep Grewal

**Affiliations:** ^1^NYIT College of Osteopathic Medicine, Old Westbury, NY, USA; ^2^Brookdale University Hospital Medical Center, Brooklyn, NY, USA

## Abstract

We describe a case of new onset angioedema likely due to Ezetimibe therapy in an elderly patient with a prior history of drug-induced bradykinin reactions who had been on the medication for multiple years. This is the second reported incidence of Ezetimibe-associated angioedema in literature. A 90-year-old African American female presented with angioedema of the face and oral mucosa with associated difficulty speaking developing hours after taking Ezetimibe 10 mg PO. She denied adding any new or unusual foods to her diet. A thorough clinical history determined Ezetimibe was the likely culprit. Ezetimibe was immediately discontinued. The swelling subsided after administration of methylprednisolone 125 mg, epinephrine 1 mg/mL, injection 0.3 mL, diphenhydramine 25 mg, and famotidine 20 mg BID within 48 hours. The patient's C1 esterase inhibitor level was measured to be within normal limits. Food panel allergy testing showed very low or undetectable IgE levels in all categories. Based on the limited reports in literature and our current case, we conclude that there is a likely association of angioedema with Ezetimibe. The mechanism, however, is unknown since it is not related to bradykinin or mast cell-mediated activation. Clinicians should advise patients taking Ezetimibe to report any swelling of the lips, face, and tongue and to immediately discontinue its use if these signs are present.

## 1. Introduction

Ezetimibe is a drug that inhibits cholesterol absorption in the small intestine, ultimately leading to lower levels of circulating cholesterol in the body [1]. In 2016, a total of 4,570,246 people were prescribed this medication, and it was ranked 144th on the list of most commonly prescribed medications in the United States [2].

Angioedema presents as nonpitting edema of the skin and/or mucous membranes, including but not limited to the gastrointestinal and respiratory tract. Numerous medications including ACE inhibitors, ARBs, and NSAIDs have been implicated in the manifestation of drug-induced angioedema [3]. Approved by the FDA in 2002 for the management of hypercholesterolemia, Ezetimibe is an uncommon culprit behind this adverse reaction. We describe an incidence of new onset angioedema likely due to Ezetimibe therapy in a patient with a past medical history of drug-induced hypersensitivity reactions.

## 2. Case Presentation

A 90-year-old African American female presented to the emergency room complaining of facial and oral swelling starting around 4:00 am that morning. She woke up and noticed her face was swollen from the right cheek to her mouth, including her lips and tongue, stating her lips were about 3 times the size from her baseline. She proceeded to splash cold water on her face and go back to sleep. At approximately 8:00 am, the patient took one dose of diphenhydramine and came to the emergency department.

The patient denied the addition of any new or unusual foods to her diet. Her last meal prior to the swelling took place 12 hours earlier at 4:00 pm. The only medication she took at night prior to bed was Ezetimibe 10 mg, which she had been taking for over 3 years. Her most recent hospital admission occurred 3 months prior due to an episode of angioedema likely related to losartan, which was promptly discontinued at that time. Her pertinent past medical history consists of arthritis, hypertension, hyperlipidemia, atrial fibrillation, and asthma. She also reported prior episodes of other drug-related hypersensitivity reactions including angiotensin receptor blocker-induced angioedema, ACE inhibitor and sacubitril-related swelling, Apixaban-related swelling, and Rosuvastatin-induced rash. Her current medications were reviewed. Following review of the Merck manual, Ezetimibe was the only drug the patient was taking which was associated with any potential risk of angioedema [4].

In the emergency department, her voice was muffled, and she continued to complain of mild difficulty with swallowing and talking. Pertinent lab values revealed an eosinophil percent of 1.4 (ref. 0.1–4.7%). Allergen CLA test of shrimp, tuna, and chicken were assigned class 0/1, indicating very low levels of IgE. The CT scan of the neck soft tissue was negative for tissue swelling. She was started on a one-time dose of epinephrine 0.3 mL intramuscular injection, methylprednisolone 125 mg IV, diphenhydramine 25 mg IV, and famotidine 20 mg IV resulting in the resolution of her symptoms within 48 hrs. C1 esterase inhibitor, C3, and C4 levels were ordered during this visit as well.

## 3. Discussion

Ezetimibe (Zetia®) is classified as an azetidinone. Unlike other lipid-lowering agents, it is part of a class of agents working in the intestines to block the uptake of cholesterol by inhibiting the Niemann–Pick C1-like 1 (NPC1L1) sterol transporter. Inhibition occurs via Ezetimibe blocking cholesterol and phytosterol uptake at the intestinal brush border, thus decreasing delivery to the liver. It is indicated in patients with hypercholesterolemia who fail to reach treatment goals on maximally tolerated statin therapy and dietary/lifestyle modifications. It is also indicated in individuals on statin therapy at high risk for recurrent atherosclerotic cardiovascular disease event. The IMPROVE-IT clinical trial revealed participants using statin therapy along with concurrent Ezetimibe use had reduced LDL cholesterol by an additional 24% and lowered risk of cardiovascular events compared with individuals on statin monotherapy [5].

Ezetimibe is predominantly metabolized in the small intestine and liver via glucuronide conjugation, producing Ezetimibe-glucuronide. Both ezetimibe and ezetimibe-glucuronide are eliminated from plasma with a half-life of approximately 22 hours. Pending FDA approval, a multiple-dose study in which Ezetimibe was given 10 mg once daily for 10 days, showed plasma concentrations for total Ezetimibe were about 2-fold higher in older (≥65 years) healthy subjects compared with younger subjects [6].

### 3.1. Angioedema: Etiology, Symptoms, and Pathophysiology

Angioedema is the swelling of areas of tissues under the skin, mucosal tissues, or both, which can occur in the face, periorbital, tongue, larynx, digestive tract, and airways. It is due to an increased permeability of submucosal capillaries with localized plasma extravasation, lasting anywhere from a few hours, up to a few days. Severe cases can be life threatening causing laryngeal edema leading to respiratory compromise. Angioedema can be subdivided into three groups of classifications by the etiology: mast cell-mediated, bradykinin-mediated, and unknown mechanism.

Mast cell-mediated angioedema is induced by histamine. It is an acute allergic reaction to foods or insect stings usually associated with urticaria, generalized pruritus, flushing, and bronchospasm. Bradykinin-mediated angioedema (nonallergic), which develops over the course of 24 to 36 hours, is due to bradykinin overproduction or inhibition of bradykinin breakdown. Mast cells are not involved; therefore, it has not been associated with urticaria, pruritus, or flushing. The most common type of bradykinin-mediated angioedema is angiotensin-converting enzyme inhibitor- (ACEI-) induced angioedema associated with a reduction in bradykinin degradation. Approximately 0.1–0.7% of patients taking this medication will experience ACEI-angioedema [7]. African Americans are at a greater risk than non-African Americans to have angioedema from angiotensin-converting enzyme (ACE) inhibitors [8]. The symptom onset can range anywhere from within a week of initiating the medication to several years after. Angioedema by etiologies of unknown mechanism includes idiopathic angioedema, infections, calcium channel blockers, and other drugs that have been reported. Sirolimus, everolimus, amiodarone, metoprolol, risperidone, paroxetine, and etanercept are amongst some of the drugs that have been reported to cause angioedema from unknown mechanism [9].

### 3.2. Angioedema Related to Ezetimibe

Within the United States, the National Hospital Ambulatory Medical Care Survey identified 108,816 (95% CI, 82,246–132,386) annual visits for angioedema in the emergency department [10]. In clinical trials, 2,396 patients on Ezetimibe monotherapy were tested, and most common reported adverse reactions (incidence ≥2% and greater than placebo) were upper respiratory tract infection (4.3%), diarrhea (4.1%), arthralgia (3.0%), sinusitis (2.8%), and pain in extremity (2.7%) [11]. There is one reported case of angioedema as a delayed reaction to Ezetimibe in 2015 by Shah et al. occurring 3 months after starting Ezetimibe [12]. In both instances, patients were taking 10 mg of Ezetimibe as directed. In postmarketing surveillance, angioedema is reported as a rare hypersensitivity reaction [11]. Despite the suspected link between angioedema and Ezetimibe, it has only been reported during phase 4 clinical trial and has not allowed its dose dependence to be determined.

As per American Academy of Allergy, Asthma, and Immunology, if a food allergy test is positive but the individual is asymptomatic when eating that specific food, there is no need to eliminate it from the diet [13]. Pinpointing Ezetimibe as the cause of our patient's angioedema was a diagnosis of exclusion. No other probable causes were identified. The reaction began approximately 12 hours after her last meal, and allergen testing results were negative, eliminating possible food derived factors. C1 esterase inhibitor levels were within normal limits at 31 mg/dL (ref. 21–39 mg/dL), excluding hereditary angioedema (HAE) as a potential cause. Patients with HAE have recurrent episodes of angioedema due to abnormal complement responses or deficiency of C1 esterase inhibitor. It is usually self-limited and does not respond to antihistamines, corticosteroids, or epinephrine. C3 complement level was 148 mg/dL, and C4 complement level was 21 mg/dL, but due to the patient falling outside of the age bracket for the reference range, values were not established.

## 4. Conclusion

Our case reports the evidence presented by Shah et al. [12] that there is an association of angioedema with the usage of Ezetimibe. Following review of the patient's clinical history and medication list, including over the counter drugs, Ezetimibe is the only drug that could have caused angioedema. Angioedema is a life-threatening hypersensitivity reaction that should be treated urgently with antihistamines, steroids, and epinephrine. It is difficult to correlate that it occurs secondary to Ezetimibe with higher incidence in the African American population or in geriatric population due to lack of sample size, while we advise that clinicians should avoid Ezetimibe in patients with a previous history of drug-induced Angioedema. When faced with any case of angioedema, the most important step is to obtain a detailed clinical history. As Ezetimibe is a relatively new FDA-approved drug, postsurveillance monitoring should be continued and reported for new cases of Ezetimibe associated angioedema.
